# Structural Elements Regulating AAA+ Protein Quality Control Machines

**DOI:** 10.3389/fmolb.2017.00027

**Published:** 2017-05-04

**Authors:** Chiung-Wen Chang, Sukyeong Lee, Francis T. F. Tsai

**Affiliations:** ^1^Verna and Marrs McLean Department of Biochemistry and Molecular Biology, Baylor College of MedicineHouston, TX, USA; ^2^Departments of Molecular and Cellular Biology, and Molecular Virology and Microbiology, Baylor College of MedicineHouston, TX, USA

**Keywords:** AAA+ proteins, protein quality control, Pre-Sensor I insert, inter-subunit signaling motif, pore loop

## Abstract

Members of the ATPases Associated with various cellular Activities (AAA+) superfamily participate in essential and diverse cellular pathways in all kingdoms of life by harnessing the energy of ATP binding and hydrolysis to drive their biological functions. Although most AAA+ proteins share a ring-shaped architecture, AAA+ proteins have evolved distinct structural elements that are fine-tuned to their specific functions. A central question in the field is how ATP binding and hydrolysis are coupled to substrate translocation through the central channel of ring-forming AAA+ proteins. In this mini-review, we will discuss structural elements present in AAA+ proteins involved in protein quality control, drawing similarities to their known role in substrate interaction by AAA+ proteins involved in DNA translocation. Elements to be discussed include the pore loop-1, the Inter-Subunit Signaling (ISS) motif, and the Pre-Sensor I insert (PS-I) motif. Lastly, we will summarize our current understanding on the inter-relationship of those structural elements and propose a model how ATP binding and hydrolysis might be coupled to polypeptide translocation in protein quality control machines.

## The AAA+ protein superfamily

**Figure 1:**
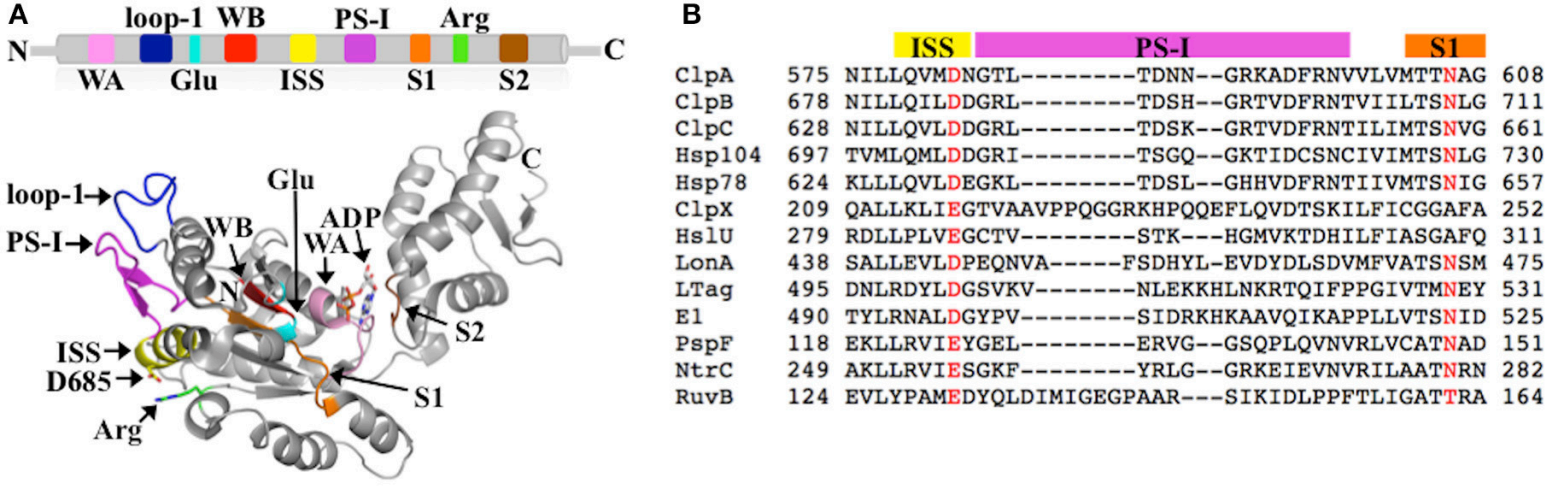
**(A)** Conserved structural elements of the PS-I superclade mapped onto the crystal structure of the ClpB-D2 domain (PDB: 4FD2) (Biter et al., 2012). Walker A motif (WA; pink), Walker B motif (WB; red), Arg-finger (Arg; green), pore loop-1 (loop-1; blue), ISS motif (yellow), PS-I β-hairpin (PS-I; magenta), sensor-1 (S1; orange), sensor-2 (S2; brown), and the glutamate-switch (Glu; cyan) (Zhang and Wigley, 2008). The same colors are used throughout in all figures. **(B)** Multiple sequence alignment of PS-I members generated using PROMALS3D (Pei et al., 2008) showing the conservation of the ISS and PS-I motifs. *Escherichia coli* ClpA; *Thermus thermophilus* ClpB; *Bacillus subtilis* ClpC; *Saccharomyces cerevisiae* Hsp104; *S. cerevisiae* Hsp78; *E. coli* ClpX; *E. coli* HslU; *E. coli* LonA; *Macaca mulatta polyomavirus* 1 Large Tumor antigen (LTag); *Deltapapillomavirus* 4 E1; *E. coli* PspF; *Aquifex aeolicus* NtrC; *E. coli* RuvB.

AAA+ proteins harness metabolic energy in form of ATP to facilitate diverse cellular processes, including organelle biogenesis, membrane fusion, transcriptional regulation, and protein quality control (PQC). Members of the AAA+ superfamily can be classified into one of four distinct clades or superclades: (1) the clamp loader clade, (2) the initiator clade, (3) the classic clade, and (4) the Pre-Sensor I insert (PS-I) superclade (Iyer et al., 2004; Erzberger and Berger, 2006). The PS-I superclade is further sub-divided into the superfamily 3 (SF3) helicase clade, the HCLR clade (HslU, ClpAB-D2, Lon, and RuvB family), the helix 2 (H2)-insert clade, and the Pre-Sensor II insert (PS-II) clade (Iyer et al., 2004; Erzberger and Berger, 2006). A hallmark of all AAA+ proteins is the AAA+ ATP-binding domain that is composed of ~220 amino acids and typically forms a hexameric ring structure in solution. The AAA+ domain features several conserved elements required for ATP binding and hydrolysis, including the Walker A and B motifs, the arginine (Arg)-finger motif, and the sensor-1 and -2 motifs (Figure 1A). In addition, each AAA+ clade features a specific insertion of a secondary structure element within the core AAA+ fold. For instance, the defining feature of the PS-I superclade is a β-hairpin insertion before the sensor-1 motif (Figures 1A,B). Despite the wealth of structural information, the functional importance of clade-specific insertions remains largely unclear.

AAA+ proteins involved in PQC include members of the Clp/Hsp100 family (Bukau et al., 2006; Olivares et al., 2016), Lon (Venkatesh et al., 2012), and FtsH-like proteases in prokaryotes and organelles (Gerdes et al., 2012; Okuno and Ogura, 2013). Clp/Hsp100 members function as protein unfoldases to facilitate either the disaggregation of previously aggregated proteins (Doyle et al., 2013; Jeng et al., 2015; Mogk et al., 2015; Sweeny and Shorter, 2016) or the degradation of ssrA-tagged proteins (Olivares et al., 2016). Members of the Clp/Hsp100 family are found in diverse microorganisms and belong to one of two classes that are distinguished by the number of AAA+ domains present in one polypeptide. Class I proteins, which include ClpA, ClpB/Hsp104 and ClpC, possess two AAA+ domains, termed the D1 and D2 domains, whereas class II proteins such as ClpX and HslU contain only a single AAA+ domain that is homologous to the second AAA+ (D2) domain of class I members (Schirmer et al., 1996). AAA+ domains assemble into a homo-hexamer composed of a D1 (class I) and a D2 ring (class I and II) that represent the physiologically active form of Clp/Hsp100 proteins. In order to facilitate protein degradation, Clp/Hsp100 proteins must associate with an oligomeric peptidase such as ClpP (Olivares et al., 2016), and assemble into a proteolytic machine of similar architecture to the 26S proteasome in Eukarya (Lee and Tsai, 2005). In contrast, PQC machines such as Lon (Venkatesh et al., 2012) and FtsH-like proteases (Gerdes et al., 2012; Okuno and Ogura, 2013) feature an integral peptidase domain that is covalently linked to the AAA+ domain.

## The pore loop-1

A hallmark of the AAA+ domain is the presence of conserved loops that line the axial channel of the oligomeric ring assembly. These pore loops have been implicated in substrate interaction. One of these pore loops, known as pore loop-1, features a Tyr/Phe- Ψ-Gly motif, where Ψ is a hydrophobic residue (Wang et al., 2001). The conserved aromatic amino acid is sensitive to mutation and was shown to impair protein function of several AAA+ ATPases when mutated (Yamada-Inagawa et al., 2003; Lum et al., 2004; Weibezahn et al., 2004). For instance, substituting the pore loop-1 tyrosine with alanine impaired substrate binding and translocation by Clp/Hsp100 proteins (Lum et al., 2004; Weibezahn et al., 2004; Hinnerwisch et al., 2005; Wang et al., 2011; Iosefson et al., 2015). The single-particle cryo-EM structure of a ClpB hexamer in the ATP-activated state showed that the D1 pore loop-1 of all six subunits is arrested at the central pore providing a platform for substrates to bind with high-affinity (Lee et al., 2007). This model is consistent with the proposed role of the D1 pore loop-1 Tyr in substrate interaction (Schlieker et al., 2004). Subsequent crystal structures of a ClpB-D2 monomer showed that pore loop-1 is stabilized by nucleotide and is mobile (i.e., disordered) in the absence of nucleotide (Biter et al., 2012; Zeymer et al., 2014), linking nucleotide binding to regulating pore loop conformation. Although the structure of a pore loop-bound substrate complex remains elusive, collectively these findings support a mechanism by which ATP-dependent changes are linked to pore loop conformations that could facilitate substrate translocation through the hexameric ring assembly.

A more recent high-resolution cryo-EM structure of yeast Hsp104 bound to AMP-PNP revealed a left-handed spiral architecture exhibiting a “staircase” arrangement of pore loops along the central channel of the Hsp104 hexamer (Yokom et al., 2016). Notably, in the cryo-EM structure the D2 domain of the 1st subunit contacts the D1 domain of the 6th subunit to give rise to a closed “lock-washer” arrangement. Although the spiral architecture is surprising, it is similar to the left-handed helical assembly observed in crystal structures of bacterial ClpB (Lee et al., 2003; Carroni et al., 2014) and a fungal Hsp104 (Heuck et al., 2016). Examining the atomic structure of a substrate-translocating Clp/Hsp100 complex will be necessary to provide direct support for the functional role of pore loops in substrate threading through the hexamer assembly.

## The ISS motif in AAA+ machines

**Figure 2:**
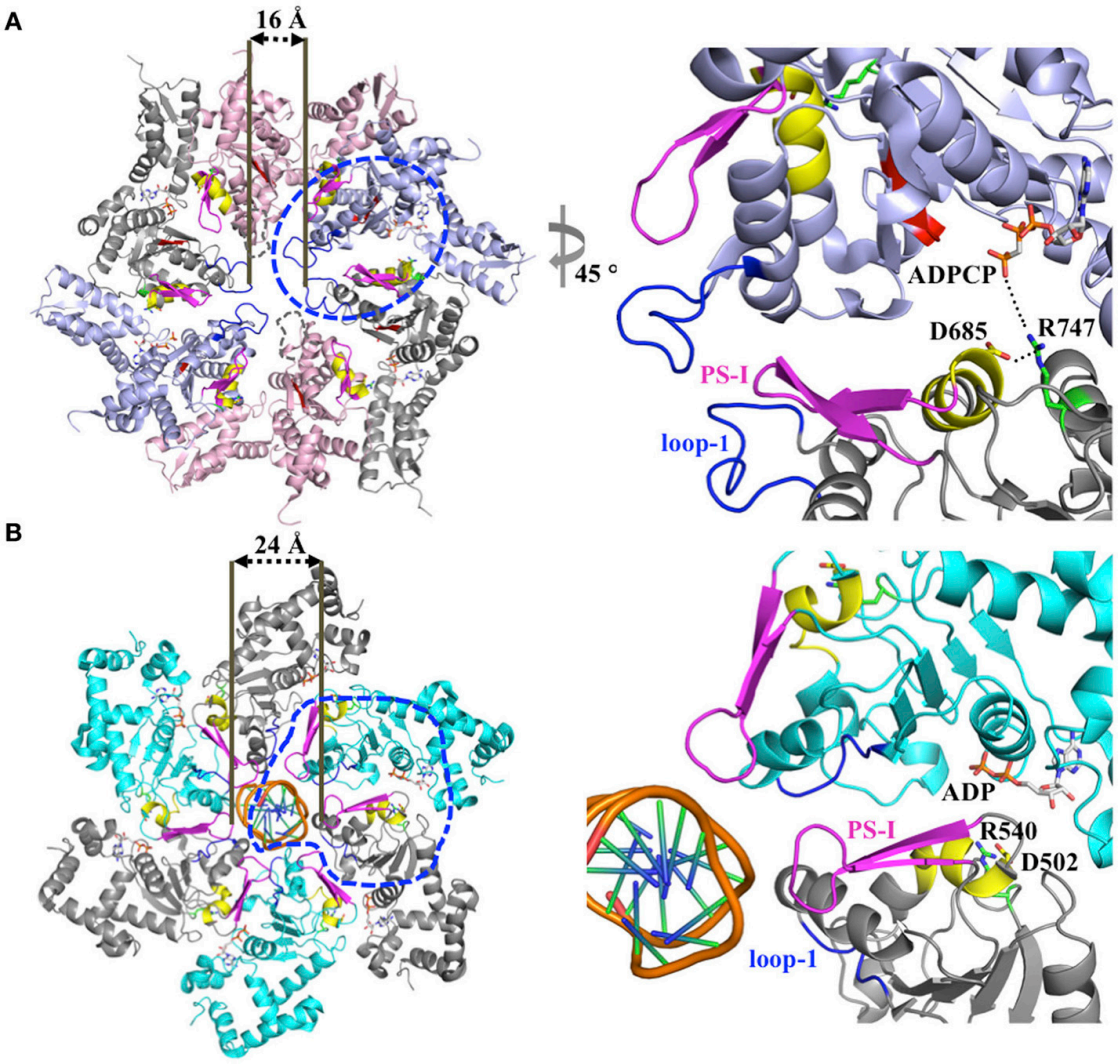
**(A)** Model for inter-subunit communication in the PS-I insert superclade of AAA+ proteins involved in PQC. Composite model based on the crystal structure of a ClpC hexamer (PDB: 3PXI) (Wang et al., 2011) following the subunit arrangement proposed by Biter et al. (2012). The hexamer model is compatible with a sequential ATP binding and hydrolysis mechanism, and consists of crystal structures of the ClpB-D2 monomer in the ATP-bound (blue, PDB: 4LJ9), ADP-bound (gray, PDB: 4FD2), nucleotide-free states (pink, PDB: 4LJ4) (Biter et al., 2012; Zeymer et al., 2014) superposed onto the ClpC-D2 large domain of the ClpC ring-shaped hexamer (Wang et al., 2011). The pore loop-1 of the ClpB-D2 domain in the nucleotide-free state, which is disordered in the available crystal structures, is indicated by a dotted line. The blue circle indicates section shown in the enlarged view. **(B)** Ribbon diagram of the SV40 LTag homo-hexamer structure bound to double-stranded DNA (PDB: 5TCT) (Gai et al., 2016). Only the helicase domains are shown. For clarity, neighboring subunits are colored differently (cyan and gray). The blue circle indicates section shown in the enlarged view.

The ISS motif consists of a network of functionally conserved residues crucial for transmitting the nucleotide status of one subunit to the adjacent subunit, thereby providing the molecular basis how ATP binding and hydrolysis is coordinated between neighboring subunits in the ring assembly. The existence of an ISS motif was first reported for the *m-*AAA protease (Augustin et al., 2009), a member of the classic clade, and is defined as the α-helix immediately preceding the sensor-1 motif featuring a characteristic aspartic or glutamic acid at its C-terminus, which interacts with a nearby arginine of the same subunit. This arginine in turn interacts with the Arg-finger that senses the nucleotide status in the adjacent subunit (Augustin et al., 2009; Hanzelmann and Schindelin, 2016). The ISS motif is also found in other members of the classic clade, including FtsH (Bieniossek et al., 2006) and p97 (Hanzelmann and Schindelin, 2016). A sequence alignment indicates that an acidic amino acid is conserved amongst members of the HCLR clade, including the D2 domain of Clp/Hsp100 proteins (Figure 1B). However, unlike members of the classic clade, the crystal structure of the ClpB-D2 domain showed a direct interaction between Asp685 and the Arg-finger (Arg747) from the same subunit (Biter et al., 2012; Zeymer et al., 2014), providing a means to directly signal the nucleotide status between neighboring subunits (Figure 2A). Consistent with a role in inter-subunit signaling, a mutation of Asp685 to alanine significantly impaired ClpB's ATPase activity (Biter et al., 2012), confirming the existence of an ISS motif in the broader AAA+ superfamily.

## The PS-I insert motif

The PS-I motif is the defining feature of members of the PS-I insert superclade (Iyer et al., 2004; Erzberger and Berger, 2006) and consists of a β-hairpin that buttresses the pore loop-1 of the same subunit (Figures 1A, 2A). Although the location of the PS-I motif is not conserved in the primary amino acid sequence of AAA+ proteins (Figure 1B), a pairwise structural comparison of different HCLR clade members shows that the location of the PS-I motif is invariant in the 3D structure. The function of the PS-I β-hairpin is perhaps best understood for AAA+ proteins involved in nucleic acid translocation, such as the simian virus 40 large tumor antigen (LTag) (Shen et al., 2005) and the papillomavirus replication initiation protein E1 (Enemark and Joshua-Tor, 2006). Structural studies of the SV40 LTag helicase bound to DNA showed that the β-hairpin is directly involved in binding to DNA (Chang et al., 2013; Gai et al., 2016). In the hexamer structure of SV40 LTag, the helicase forms a near-planar ring with the β-hairpin lining the inner surface of the central channel encircling the double-stranded DNA helix (Gai et al., 2016) (Figure 2B). Substrate contacts are mediated by a combination of hydrogen bonding, electrostatic and hydrophobic interactions between residues at the tip of the β-hairpin (Lys512 and His513) and the phosphate backbone, the sugar moieties and the edges of bases of the DNA (Chang et al., 2013; Gai et al., 2016). It has been suggested that ATP-driven domain motions are transmitted to the β-hairpin resulting in DNA translocation along the central channel (Gai et al., 2004; Chang et al., 2013). The importance of the PS-I β-hairpin in substrate binding is also supported by the crystal structure of a hexameric E1 helicase bound to a single-strand of DNA (Enemark and Joshua-Tor, 2006). Consistent with a potential role of the PS-I hairpin in substrate binding, deletion of the β-hairpin loop in ClpB (ClpB_Δ691–695_) impaired protein disaggregation to similar levels to that observed with a ClpB variant featuring a D2 pore loop tyrosine to alanine mutation (ClpB_Y643A_) (Biter et al., 2012). Although the ATPase activity is also reduced, it is similar for both mutants (Biter et al., 2012).

More recently, the crystal structure of a fungal Hsp104 in the ADP-bound state was determined (Heuck et al., 2016) revealing a different β-hairpin conformation that contacts the D1 domain, and is distinct from the β-hairpin conformation seen in crystal structures of bacterial ClpB (Lee et al., 2003; Biter et al., 2012; Carroni et al., 2014; Zeymer et al., 2014) and in the aforementioned helicases (Enemark and Joshua-Tor, 2006; Gai et al., 2016). Although deletion of the PS-I insert motif significantly impaired the Hsp104 protein disaggregating activity (Heuck et al., 2016), the interpretation of the observed defect is different. In the case of Hsp104, it was proposed that the PS-I insert motif is involved in signaling the nucleotide status between the two AAA+ rings and is responsible for allosteric regulation that controls Hsp104 function (Franzmann et al., 2011; Heuck et al., 2016). Although not mutually exclusive, determining the functional importance of the PS-I motif in ClpB/Hsp104 chaperones requires further structural and biochemical confirmation.

## Coupling the ATPase cycle to substrate translocation in PQC machines

The available 3D structures of AAA+ machines involved in PQC have provided snapshots of distinct functional states and have contributed toward our molecular understanding how the ATPase cycle is coupled to conformational changes needed for substrate translocation. Structural evidence suggests that the pore loop-1 conformation optimized for substrate binding is determined by the nucleotide-bound status of the *cis*-subunit, which in turn is controlled by the nucleotide state of the *trans*-subunit (Biter et al., 2012) (Figure 2A). In this model, the Arg-finger of the *cis*-subunit senses the ATP-bound state in the neighboring subunit and transmits this signal in *cis* via a conserved acidic amino acid residue (either Asp or Glu) of the ISS motif, triggering ATP hydrolysis in the *cis*-subunit concomitant with substrate translocation. We propose that the PS-I motif communicates with pore loop-1 and controls substrate interaction by either contacting the substrate directly or regulating the ATPase cycle in the D2 ring through communication with the D1 ring. Although the available structural and biochemical evidence provide support for such mechanism, determining the structure of a substrate bound complex will be necessary to provide a more accurate mechanistic understanding how the ATPase cycle is coupled to substrate translocation in PQC machines.

## Author contributions

CC, SL, and FT contributed to writing the draft and final version of this mini-review.

## Funding

Research in the FT and SL laboratories is supported by grants from the National Institutes of Health (GM104980 and GM111084) and the Welch Foundation (Q-1530). CC is the recipient of an American Heart Association-Southwest Affiliate Postdoctoral Fellowship.

### Conflict of interest statement

The authors declare that the research was conducted in the absence of any commercial or financial relationships that could be construed as a potential conflict of interest.

## References

[B1] Augustin S. Gerdes F. Lee S. Tsai F. T. Langer T. Tatsuta T. (2009). An intersubunit signaling network coordinates ATP hydrolysis by *m*-AAA proteases. Mol. Cell 35, 574–585. 10.1016/j.molcel.2009.07.018 19748354 PMC2744646

[B2] Bieniossek C. Schalch T. Bumann M. Meister M. Meier R. Baumann U. (2006). The molecular architecture of the metalloprotease FtsH. Proc. Natl. Acad. Sci. U.S.A. 103, 3066–3071. 10.1073/pnas.0600031103 16484367 PMC1413944

[B3] Biter A. B. Lee S. Sung N. Tsai F. T. (2012). Structural basis for intersubunit signaling in a protein disaggregating machine. Proc. Natl. Acad. Sci. U.S.A. 109, 12515–12520. 10.1073/pnas.1207040109 22802670 PMC3411974

[B4] Bukau B. Weissman J. Horwich A. (2006). Molecular chaperones and protein quality control. Cell 125, 443–451. 10.1016/j.cell.2006.04.014 16678092

[B5] Carroni M. Kummer E. Oguchi Y. Wendler P. Clare D. K. Sinning I.. (2014). Head-to-tail interactions of the coiled-coil domains regulate ClpB activity and cooperation with Hsp70 in protein disaggregation. Elife 3:e02481. 10.7554/eLife.02481 24843029 PMC4023160

[B6] Chang Y. P. Xu M. Machado A. C. Yu X. J. Rohs R. Chen X. S. (2013). Mechanism of origin DNA recognition and assembly of an initiator-helicase complex by SV40 large tumor antigen. Cell Rep. 3, 1117–1127. 10.1016/j.celrep.2013.03.002 23545501 PMC3748285

[B7] Doyle S. M. Genest O. Wickner S. (2013). Protein rescue from aggregates by powerful molecular chaperone machines. Nat. Rev. Mol. Cell Biol. 14, 617–629. 10.1038/nrm3660 24061228

[B8] Enemark E. J. Joshua-Tor L. (2006). Mechanism of DNA translocation in a replicative hexameric helicase. Nature 442, 270–275. 10.1038/nature04943 16855583

[B9] Erzberger J. P. Berger J. M. (2006). Evolutionary relationships and structural mechanisms of AAA+ proteins. Annu. Rev. Biophys. Biomol. Struct. 35, 93–114. 10.1146/annurev.biophys.35.040405.101933 16689629

[B10] Franzmann T. M. Czekalla A. Walter S. G. (2011). Regulatory circuits of the AAA+ disaggregase Hsp104. J. Biol. Chem. 286, 17992–18001. 10.1074/jbc.M110.216176 21454552 PMC3093873

[B11] Gai D. Wang D. Li S. X. Chen X. S. (2016). The structure of SV40 large T hexameric helicase in complex with AT-rich origin DNA. Elife 5:e18129. 10.7554/eLife.18129 27921994 PMC5140265

[B12] Gai D. Zhao R. Li D. Finkielstein C. V. Chen X. S. (2004). Mechanisms of conformational change for a replicative hexameric helicase of SV40 large tumor antigen. Cell 119, 47–60. 10.1016/j.cell.2004.09.017 15454080

[B13] Gerdes F. Tatsuta T. Langer T. (2012). Mitochondrial AAA proteases–towards a molecular understanding of membrane-bound proteolytic machines. Biochim. Biophys. Acta 1823, 49–55. 10.1016/j.bbamcr.2011.09.015 22001671

[B14] Hanzelmann P. Schindelin H. (2016). Structural Basis of ATP Hydrolysis and Intersubunit Signaling in the AAA+ ATPase p97. Structure 24, 127–139. 10.1016/j.str.2015.10.026 26712278

[B15] Heuck A. Schitter-Sollner S. Suskiewicz M. J. Kurzbauer R. Kley J. Schleiffer A.. (2016). Structural basis for the disaggregase activity and regulation of Hsp104. Elife 5:e21516. 10.7554/eLife.21516 27901467 PMC5130295

[B16] Hinnerwisch J. Fenton W. A. Furtak K. J. Farr G. W. Horwich A. L. (2005). Loops in the central channel of ClpA chaperone mediate protein binding, unfolding, and translocation. Cell 121, 1029–1041. 10.1016/j.cell.2005.04.012 15989953

[B17] Iosefson O. Nager A. R. Baker T. A. Sauer R. T. (2015). Coordinated gripping of substrate by subunits of a AAA+ proteolytic machine. Nat. Chem. Biol. 11, 201–206. 10.1038/nchembio.1732 25599533 PMC4333055

[B18] Iyer L. M. Leipe D. D. Koonin E. V. Aravind L. (2004). Evolutionary history and higher order classification of AAA+ ATPases. J. Struct. Biol. 146, 11–31. 10.1016/j.jsb.2003.10.010 15037234

[B19] Jeng W. Lee S. Sung N. Lee J. Tsai F. T. (2015). Molecular chaperones: guardians of the proteome in normal and disease states. F1000Res. 4:1448. 10.12688/f1000research.7214.1 26918154 PMC4754035

[B20] Lee S. Choi J. M. Tsai F. T. (2007). Visualizing the ATPase cycle in a protein disaggregating machine: structural basis for substrate binding by ClpB. Mol. Cell 25, 261–271. 10.1016/j.molcel.2007.01.002 17244533 PMC1855157

[B21] Lee S. Sowa M. E. Watanabe Y. Sigler P. B. Chiu W. Yoshida M.. (2003). The structure of ClpB: a molecular chaperone that rescues proteins from an aggregated state. Cell 115, 229–240. 10.1016/S0092-8674(03)00807-9 14567920

[B22] Lee S. Tsai F. T. (2005). Molecular chaperones in protein quality control. J. Biochem. Mol. Biol. 38, 259–265. 10.5483/bmbrep.2005.38.3.259 15943899

[B23] Lum R. Tkach J. M. Vierling E. Glover J. R. (2004). Evidence for an unfolding/threading mechanism for protein disaggregation by *Saccharomyces cerevisiae* Hsp104. J. Biol. Chem. 279, 29139–29146. 10.1074/jbc.M403777200 15128736

[B24] Mogk A. Kummer E. Bukau B. (2015). Cooperation of Hsp70 and Hsp100 chaperone machines in protein disaggregation. Front. Mol. Biosci. 2:22. 10.3389/fmolb.2015.00022 26042222 PMC4436881

[B25] Okuno T. Ogura T. (2013). FtsH protease-mediated regulation of various cellular functions. Subcell. Biochem. 66, 53–69. 10.1007/978-94-007-5940-4_3 23479437

[B26] Olivares A. O. Baker T. A. Sauer R. T. (2016). Mechanistic insights into bacterial AAA+ proteases and protein-remodelling machines. Nat. Rev. Microbiol. 14, 33–44. 10.1038/nrmicro.2015.4 26639779 PMC5458636

[B27] Pei J. Kim B. H. Grishin N. V. (2008). PROMALS3D: a tool for multiple protein sequence and structure alignments. Nucleic Acids Res. 36, 2295–2300. 10.1093/nar/gkn072 18287115 PMC2367709

[B28] Schirmer E. C. Glover J. R. Singer M. A. Lindquist S. (1996). HSP100/Clp proteins: a common mechanism explains diverse functions. Trends Biochem. Sci. 21, 289–296. 10.1016/S0968-0004(96)10038-4 8772382

[B29] Schlieker C. Weibezahn J. Patzelt H. Tessarz P. Strub C. Zeth K.. (2004). Substrate recognition by the AAA+ chaperone ClpB. Nat. Struct. Mol. Biol. 11, 607–615. 10.1038/nsmb787 15208691

[B30] Shen J. Gai D. Patrick A. Greenleaf W. B. Chen X. S. (2005). The roles of the residues on the channel beta-hairpin and loop structures of simian virus 40 hexameric helicase. Proc. Natl. Acad. Sci. U.S.A. 102, 11248–11253. 10.1073/pnas.0409646102 16061814 PMC1183535

[B31] Sweeny E. A. Shorter J. (2016). Mechanistic and Structural insights into the prion-disaggregase activity of Hsp104. J. Mol. Biol. 428, 1870–1885. 10.1016/j.jmb.2015.11.016 26608812 PMC4860052

[B32] Venkatesh S. Lee J. Singh K. Lee I. Suzuki C. K. (2012). Multitasking in the mitochondrion by the ATP-dependent Lon protease. Biochim. Biophys. Acta 1823, 56–66. 10.1016/j.bbamcr.2011.11.003 22119779 PMC3263341

[B33] Wang F. Mei Z. Qi Y. Yan C. Hu Q. Wang J.. (2011). Structure and mechanism of the hexameric MecA-ClpC molecular machine. Nature 471, 331–335. 10.1038/nature09780 21368759

[B34] Wang J. Song J. J. Franklin M. C. Kamtekar S. Im Y. J. Rho S. H.. (2001). Crystal structures of the HslVU peptidase-ATPase complex reveal an ATP-dependent proteolysis mechanism. Structure 9, 177–184. 10.1016/S0969-2126(01)00570-6 11250202

[B35] Weibezahn J. Tessarz P. Schlieker C. Zahn R. Maglica Z. Lee S.. (2004). Thermotolerance requires refolding of aggregated proteins by substrate translocation through the central pore of ClpB. Cell 119, 653–665. 10.1016/j.cell.2004.11.027 15550247

[B36] Yamada-Inagawa T. Okuno T. Karata K. Yamanaka K. Ogura T. (2003). Conserved pore residues in the AAA protease FtsH are important for proteolysis and its coupling to ATP hydrolysis. J. Biol. Chem. 278, 50182–50187. 10.1074/jbc.M308327200 14514680

[B37] Yokom A. L. Gates S. N. Jackrel M. E. Mack K. L. Su M. Shorter J.. (2016). Spiral architecture of the Hsp104 disaggregase reveals the basis for polypeptide translocation. Nat. Struct. Mol. Biol. 23, 830–837. 10.1038/nsmb.3277 27478928 PMC5509435

[B38] Zeymer C. Barends T. R. Werbeck N. D. Schlichting I. Reinstein J. (2014). Elements in nucleotide sensing and hydrolysis of the AAA+ disaggregation machine ClpB: a structure-based mechanistic dissection of a molecular motor. Acta Crystallogr. D Biol. Crystallogr. 70, 582–595. 10.1107/S1399004713030629 24531492 PMC3940203

[B39] Zhang X. Wigley D. B. (2008). The 'glutamate switch' provides a link between ATPase activity and ligand binding in AAA+ proteins. Nat. Struct. Mol. Biol. 15, 1223–1227. 10.1038/nsmb.1501 18849995 PMC2806578

